# Supplementary material for: Towards an in-depth characterization of Symbiodiniaceae in tropical giant clams via metabarcoding of pooled multi-gene amplicons
Source: PeerJ. 2019 May 13;7:e6898. doi: 10.7717/peerj.6898 (PMC6521813; doi:10.7717/peerj.6898)
Supplement: Supplemental Information 1 — Identification numbers, collection localities and date collected for the twelve samples of Tridacna maxima investigated in this study. [file peerj-07-6898-s001.docx]

**Table S1** Identification numbers, collection localities and date collected for the twelve samples of *Tridacna maxima* investigated in this study.

| **Sample ID** | **Collection locality** | **Latitude** | **Longitude** | **Date collected (Year/Month)** |
| --- | --- | --- | --- | --- |
| S141 | Raivavae frangeant (Australes) | 23°51.550 | 147°41.046 | 2013/04 |
| S142 | Mahina pointe Venus (Tahiti) | 17°31.05 | 149°30.34 | 2013/02 |
| S143 | Mangareva baie de Gatavake (Gambiers) | 23°06.51 | 134°59.32 | 2012/05 |
| S144 | Kauehi (Tuamotu) | 15°49.05 | 145°6.54 | 2013/05 |
| S145 | Kaukura (Tuamotu) | 15°38.556 | 146°49.507 | 2012/* |
| S146 | Makemo (Tuamotu) | 16°37.322 | 143°36.298 | 2011/03 |
| S147 | Papara (Tahiti) | 17°45.816 | 149°30.4 | 2011/02 |
| S148 | Tubuai barrière (Australes) | 23°24.789 | 149°27.219 | 2013/04 |
| S149 | Raivavae Motu Mano (Australes) | 23°53.47 | 147°41.06 | 2012/04 |
| S150 | Lafayette (Tahiti) | 17°31.25 | 149°31.16 | 2013/02 |
| S151 | Tikehau (Tuamotu) | 15°06.19 | 148°12.00 | 2013/05 |
| S152 | Maria (Australes) | 21°47.407 | 154°42.221 | 2013/04 |
|  |  |  |  |  |
| * Month of collection not available | | | | |
