# Supplementary material for: Towards an in-depth characterization of Symbiodiniaceae in tropical giant clams via metabarcoding of pooled multi-gene amplicons
Source: PeerJ. 2019 May 13;7:e6898. doi: 10.7717/peerj.6898 (PMC6521813; doi:10.7717/peerj.6898)
Supplement: Supplemental Information 2 — List of primers used for generating PCR amplicons. Illumina adaptors are shown in bold. [file peerj-07-6898-s002.docx]

**Table S2** List of primers used for generating PCR amplicons. Illumina adaptors are shown in bold.

| **Primer Name** | **Forward/Reverse** | **length** | **Sequence** | **Melting Temp. ° C** |
| --- | --- | --- | --- | --- |
| ITSD_illu | Fwd | 53 | 5' - **TCG TCG GCA GCG TCA GAT GTG TAT AAG AGA CAG** GTG AAT TGC AGA ACT CCG TG - 3' | 69.8 |
| ITS2Rev2_illu | Rev | 55 | 5' - **GTC TCG TGG GCT CGG AGA TGT GTA TAA GAG ACA** GCC TCC GCT TAC TTA TAT GCT T - 3' | 68.9 |
| 23SHyperUP_illu | Fwd | 53 | 5' - **TCG TCG GCA GCG TCA GAT GTG TAT AAG AGA CAG** TCA GTA CAA ATA ATA TGC TG - 3' | 66.7 |
| 23SHyperDN_illu | Rev | 55 | 5' - **GTC TCG TGG GCT CGG AGA TGT GTA TAA GAG ACA** GTT ATC GCC CCA ATT AAA CAG T -3' | 68.5 |
| LSU1F_illu | Fwd | 52 | 5' - **TCG TCG GCA GCG TCA GAT GTG TAT AAG AGA CAG** GCG GAG GAA AAG RAA CTA A - 3' | 69.4 |
| LSU1R_illu | Rev | 54 | 5' - **GTC TCG TGG GCT CGG AGA TGT GTA TAA GAG ACA** GGT CCT TTT CAT CTT TCC CTC - 3' | 68.6 |
