# Supplementary material for: Towards an in-depth characterization of Symbiodiniaceae in tropical giant clams via metabarcoding of pooled multi-gene amplicons
Source: PeerJ. 2019 May 13;7:e6898. doi: 10.7717/peerj.6898 (PMC6521813; doi:10.7717/peerj.6898)

A) Forward LSU primer (LSU1F_illu: 5’- GCG GAG GAA AAG RAA CTA A -3')

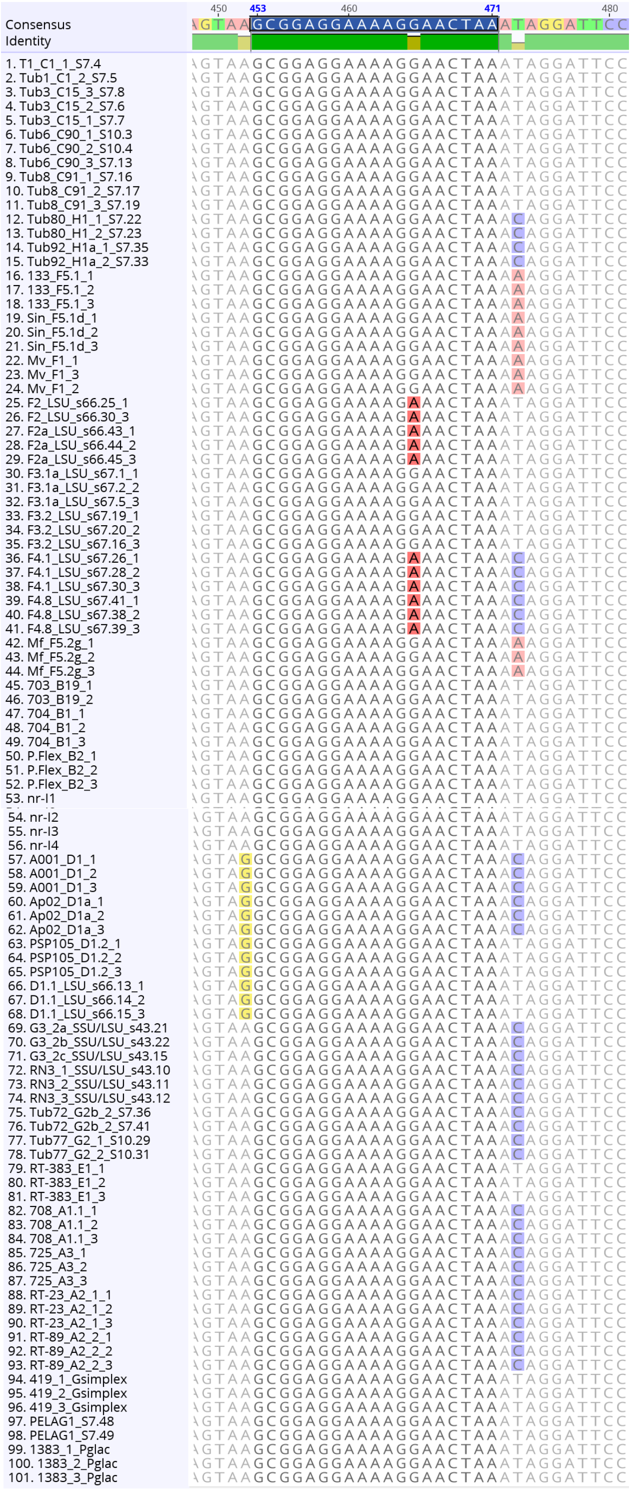

B) Reverse LSU primer (LSU1R_illu 5’- AGT CCT TTT CAT CTT TCC CTC -3')

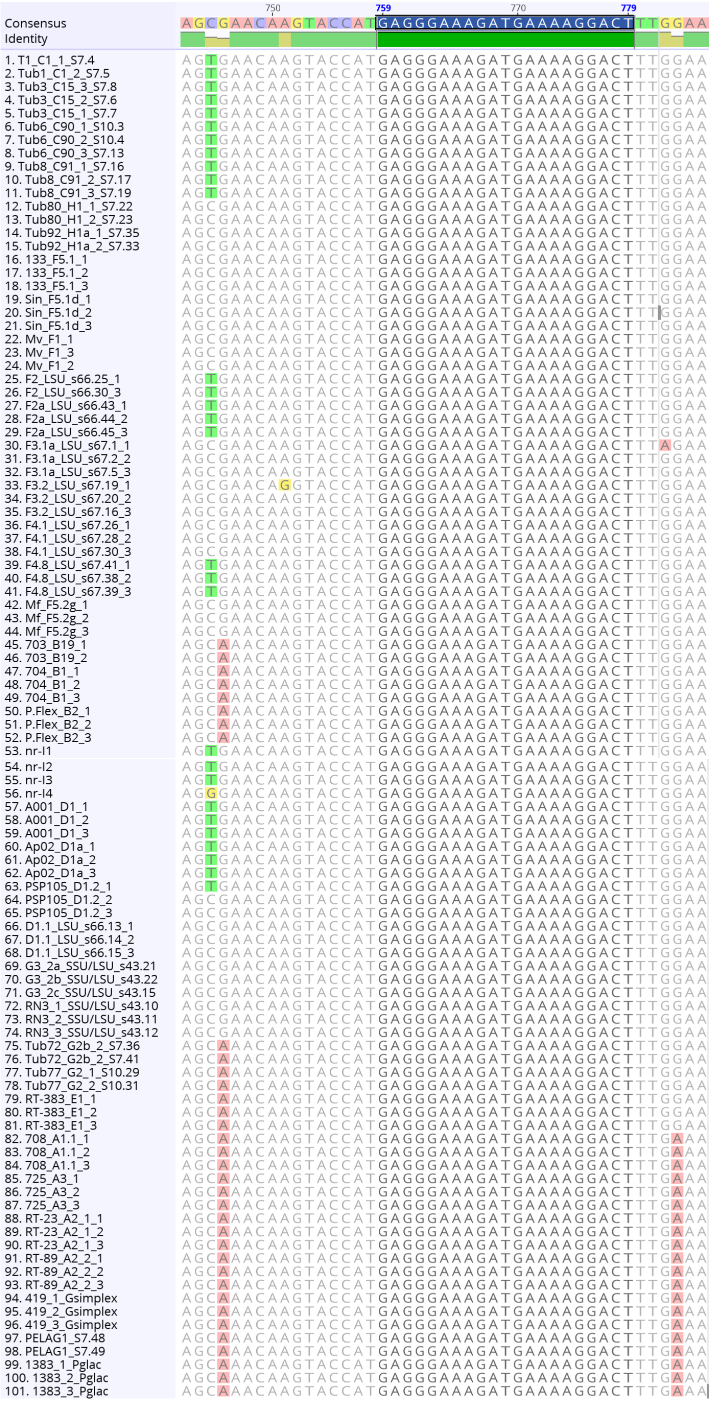

Supplement: Supplemental Information 4 — Location of the forward (LSU1F_illu; base-pair position 453–471) and reverse (LSU1R_illu; base-pair position 759–779) LSU primers based on the 1,340 bp sequence alignment (ITS2 and LSU) published in Pochon et al., 2012 (Full fasta alignment provided in the File S1). [file peerj-07-6898-s004.pdf]
