# Supplementary material for: Towards an in-depth characterization of Symbiodiniaceae in tropical giant clams via metabarcoding of pooled multi-gene amplicons
Source: PeerJ. 2019 May 13;7:e6898. doi: 10.7717/peerj.6898 (PMC6521813; doi:10.7717/peerj.6898)
Supplement: Supplemental Information 5 — Unrooted circled trees of Symbiodiniaceae genotypes inferred using the Neighbor-Joining method, with (A) 11 23S sequences, (B) 46 ITS2 sequences, and (C) 51 LSU sequences. [file peerj-07-6898-s005.pdf]

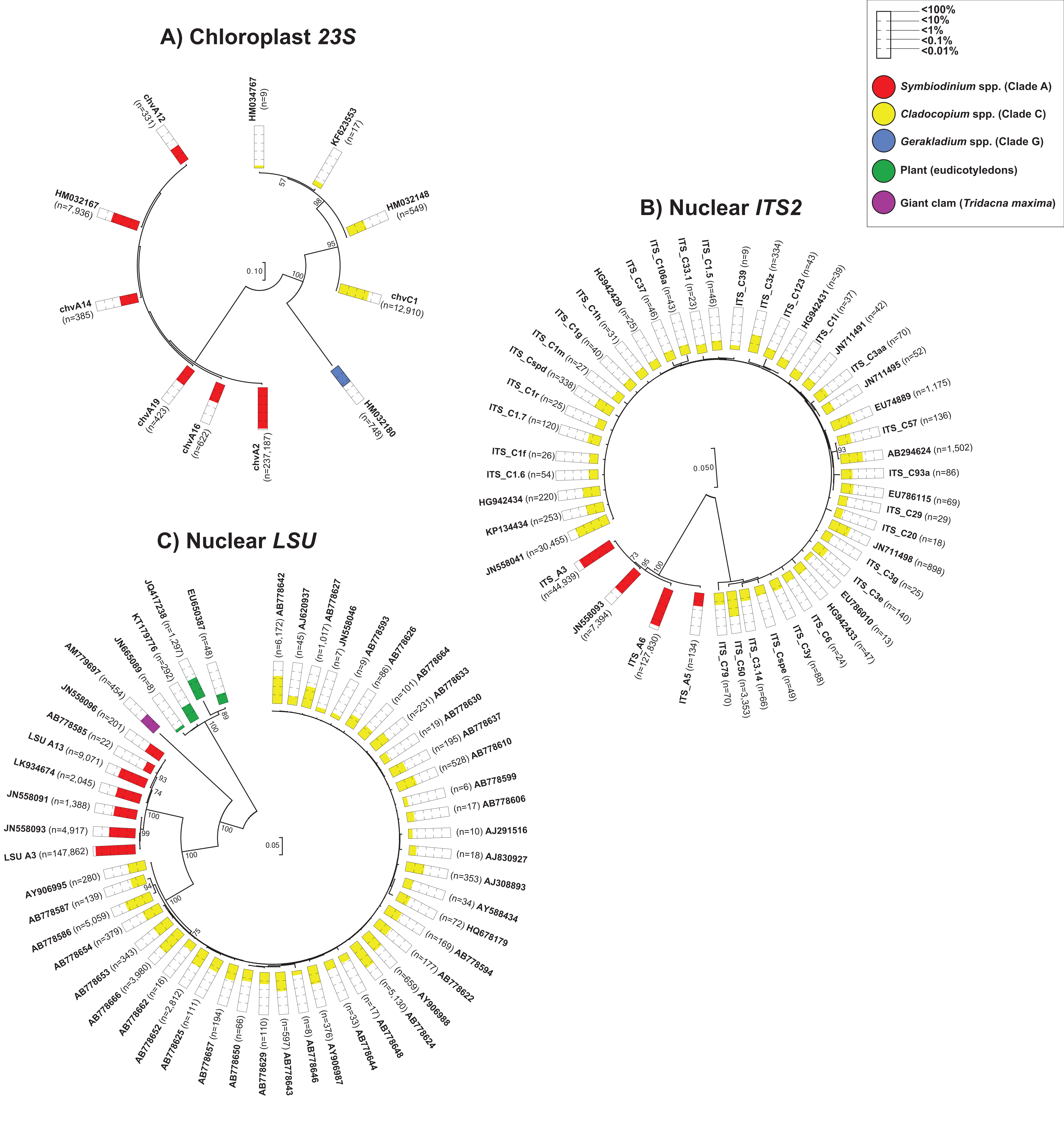

Figure S1 Unrooted circled trees of Symbiodiniaceae genotypes inferred using the Neighbor-Joining method, with (A) 11 23S sequences, (B) 46 ITS2 sequences, and (C) 51 LSU sequences. The trees aim to visualize the pair-wise relationships and sequence abundance of Symbiodiniaceae genotypes recovered from the twelve giant clam samples and among the three investigated genes. Scale bars at center of trees correspond to the number of changes per site. Numbers at nodes are the bootstrap values >50%. The colorful bubbles represents the abundance of a given genotype with the size being proportional to the total number of sequences retained per gene (log10 transformed; see internal inset scale).
