# Supplementary material for: Towards an in-depth characterization of Symbiodiniaceae in tropical giant clams via metabarcoding of pooled multi-gene amplicons
Source: PeerJ. 2019 May 13;7:e6898. doi: 10.7717/peerj.6898 (PMC6521813; doi:10.7717/peerj.6898)
Supplement: Supplemental Information 6 — Distribution of Symbiodiniaceae genera (i.e., clades) in Tridacna maxima obtained from each of the three datasets (left to right: 23S, ITS2, and LSU) per sample identification (S141–152). [file peerj-07-6898-s006.pdf]

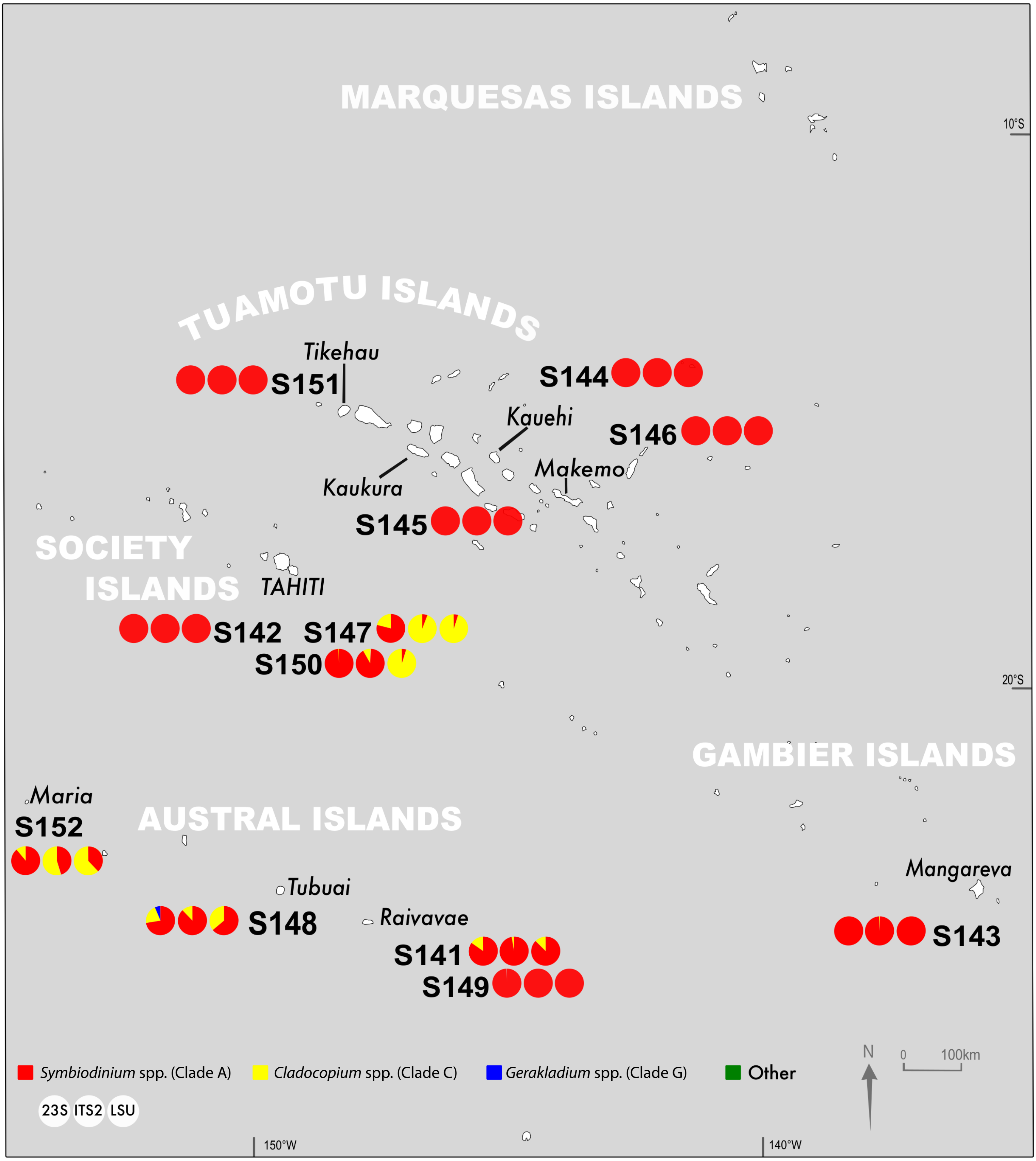

Figure S2 Distribution of Symbiodiniaceae genera in *Tridacna maxima* obtained from each of the three datasets (left to right: 23S, ITS2, and LSU) per sample identification (S141-152).
